# Genome Sequences of Equine Herpesvirus 1 Strains from a European Outbreak of Neurological Disorders Linked to a Horse Gathering in Valencia, Spain, in 2021

**DOI:** 10.1128/MRA.00333-21

**Published:** 2021-05-20

**Authors:** Nick Vereecke, Flora Carnet, Stéphane Pronost, Katleen Vanschandevijl, Sebastiaan Theuns, Hans Nauwynck

**Affiliations:** a Department Virology, Parasitology, and Immunology, Faculty of Veterinary Medicine, Ghent University, Merelbeke, Belgium; b PathoSense BV, Lier, Belgium; c LABÉO Frank Duncombe, Saint-Contest, France; d Normandie University, Unicaen, Biotargen, Caen, France; e Dierenkliniek de Bosdreef, Moerbeke-Waas, Belgium; Queens College CUNY

## Abstract

Five equine herpesvirus 1 (EHV-1) genome sequences with links to an EHV-1 outbreak with neurological disorders after a horse gathering in Valencia, Spain, in February 2021, were determined. All strains showed the closest relationships to strains from Belgium and the United Kingdom, indicating a common source of infection.

## ANNOUNCEMENT

One of the most serious equine herpesvirus 1 (EHV-1) outbreaks in Europe was reported following the International CES Valencia Spring Tour (Spain) in February 2021, which was attended by 752 horses. As 17 dead horses and neurological disorders were reported, quarantine regulations were implemented quickly in Spain and other European Union countries to prevent further spread (19 March 2021). Nevertheless, the aggressive EHV-1 strain escaped Spain with outbreaks in nine countries, including Belgium and France (FEI Updates 2021, https://inside.fei.org/fei/ehv-1/department-updates?year=). Here, we report five genomes from EHV-1 isolates from affected horses in Belgium and France with links to the Spanish Tour in 2021, as obtained through rapid long-read sequencing.

EHV-1 was isolated on rabbit kidney (RK-13) cells from nasal swab samples or peripheral blood mononuclear cells (PBMCs) from Belgian and French horses with neurological symptoms after attending the Spanish Sunshine Tour in February 2021 (Table 1).

**TABLE 1:** Overview of clinical data and sequencing output for three Belgian and two French EHV-1 isolates

Strain	Origin	Horse age (yr)	Horse gender^*a*^	Horse type	Site in Spain (sampling time^*b*^)	PCR test result for^*c*^:		Virus isolation from:		Sequencing output			
						Nose sample	Blood sample	Nose sample	PBMCs	Length (bp)	GC content (%)	Coverage (×)	Read *N*_50_ (bp)
BE/21P40/2021	Belgium	8	F	Jumping	Valencia (2 wk)	+	+	+	−	149,513	56.6	230	4,058
BE/21P41/2021	Belgium	12	F	Jumping	Vejer (<24 h)	+	+	+	+	154,163	56.5	140	4,169
BE/21P43_BD5/2021	Belgium	11	F	Jumping	Valencia (2 wk)	+	+	−	+	161,236	56.6	592	1,748
FR/Valencia1/2021	France	6	F	Jumping	Valencia (<24 h)	+	ND	+	ND	150,964	56.6	311	400
RF/Valencia2/2021	France	9	F	Jumping	Valencia (<24 h)	+	ND	+	ND	156,482	56.5	309	417

a F, female.

b The sampling time indicated is the time between the return from Spain and sampling.

c +, positive result; −, negative result; ND, not determined (test was not performed).

Viral DNA was extracted from the cell culture supernatant using the Quick-DNA/RNA viral kit (Zymo Research) at PathoSense BV. Native EHV-1 DNA was sequenced on a MinION R.9.4 flow cell (FLO-MIN106; Oxford Nanopore Technologies [ONT]) using the ONT ligation sequencing protocol (LSK-109; ONT). Raw data were processed using an in-house pipeline. In short, raw data were base called using the high-accuracy algorithm, demultiplexed and trimmed, and filtered using Guppy (v3.6; ONT) (-c dna_r9.4.1_450bps_hac.cfg), qcat (v1.1.0; ONT), and NanoFilt (v2.7.1) (1), respectively. Filtered reads were used for *de novo* EHV-1 genome assembly using Canu (v2.0) (2). Consensus genomes were obtained after read alignment using GraphMap (v0.5.2) (3) and polishing using medaka (v1.0.0; ONT). Downstream analyses included multiple-sequence alignment, pairwise identity determination, and phylogenetic analysis using MAFFT (v7.471) (4), BLASTN (v2.10.1+), and FastTree (v2) (5) (-nt -gamma -gtr), respectively. All software was run using default settings. Values are represented as means ± standard deviation.

**FIG 1:**
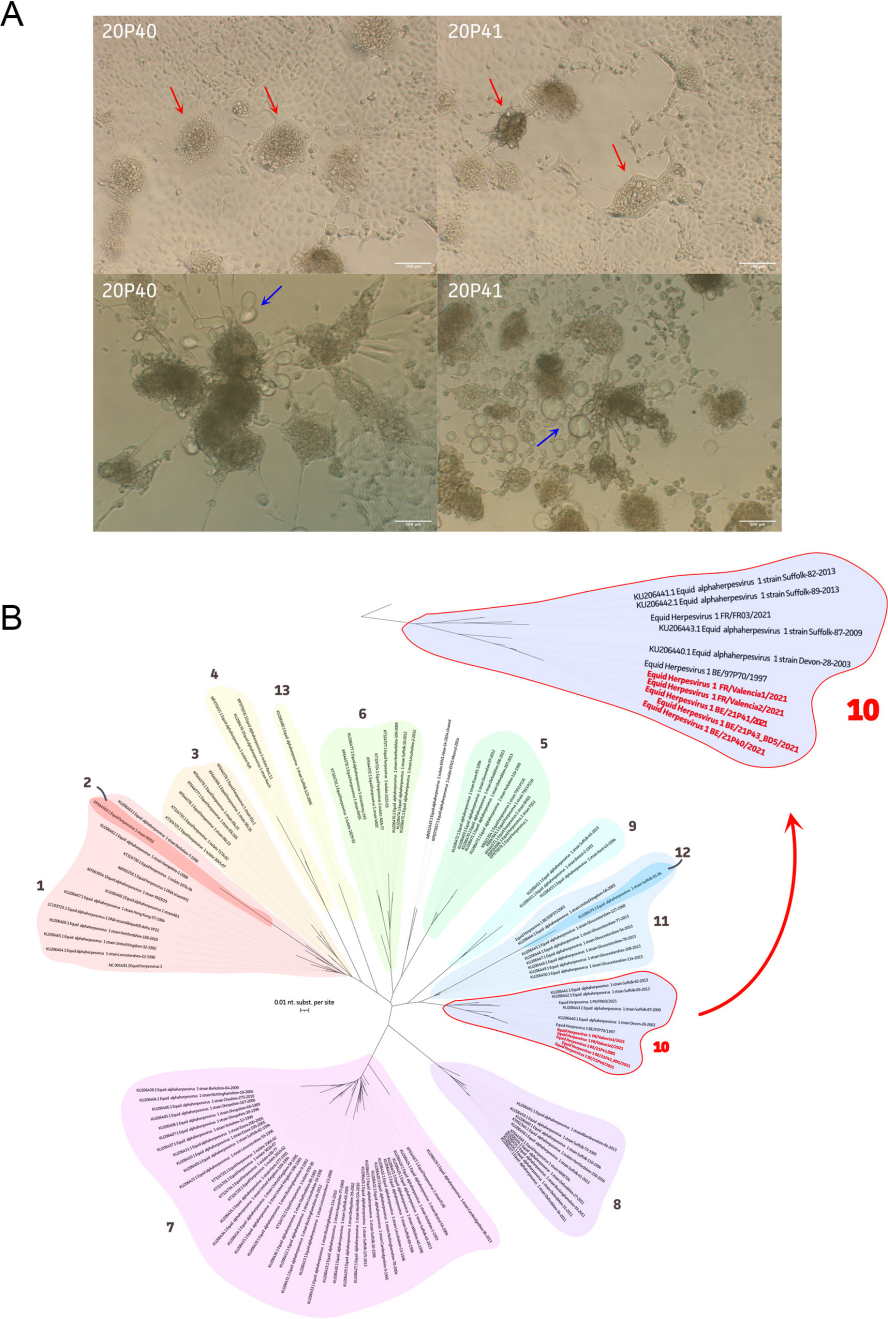
Phenotypic characterization and clade determination of new EHV-1 strains. (A) Phenotypic characterization of two Belgian isolates (21P40 and 21P41) on RK-13 cells, showing the formation of syncytia (red arrows) and balloon-shaped structures (blue arrows). Bar, 500 μm. (B) Phylogenetic tree of all available EHV-1 genomes, highlighting the new Belgian (*n* = 3) and French (*n* = 2) EHV-1 strains in red within clade 10.

The three Belgian isolates showed comparable cytopathic effect (CPE) on RK-13 cells. As shown in Fig. 1A, formation of typical EHV-1 syncytia was observed. Multiple balloon-shaped structures were present close to these syncytia.

The EHV-1 genomes (154,472 ± 4,658 bp) were phylogenetically classified based on clades previously defined by Bryant et al. (6). All Belgian and French outbreak strains were closely related to each other (nucleotide identities of 99.91 ± 0.03%) and belonged to clade 10. This suggests the spread of a single EHV-1 strain during the outbreak in Spain. Interestingly, clade 10 comprised a total of four abortive EHV-1 strains from Belgium (*n* = 1; 1997) and the United Kingdom (*n* = 3; 2009 to 2013) (Fig. 1B). We further analyzed whether G2254/D752, among other neuropathogenic markers, was present in open reading frame 30 (ORF30) (6–8). All isolates demonstrated an H250/N752/Y753/K990 genotype. In the past, the N752 (A2254) marker was shown to be predominant (52% to 97%) in abortion cases in different countries (8–13). While mostly partial genomes are available (<80% nucleotides of ∼150 kbp), we encourage more complete high-quality genome sequences and clinical data to be made available in order to establish a clear genetic EHV-1 context (6, 8).

### Data availability.

The EHV-1 genome sequences were deposited in the NCBI database, and raw reads (Nanopore) are available in the ENA (BioProject number PRJEB43980). The accession numbers are MW855958 (BE/21P40/2021), MW855959 (BE/21P41/2021), MW855960 (BE/21P43_BD5/2021), MW855961 (FR/Valencia1/2021), and MW855962 (FR/Valencia2/2021).
